# Supervised filters for EEG signal in naturally occurring epilepsy forecasting

**DOI:** 10.1371/journal.pone.0178808

**Published:** 2017-06-20

**Authors:** Francisco Javier Muñoz-Almaraz, Francisco Zamora-Martínez, Paloma Botella-Rocamora, Juan Pardo

**Affiliations:** ESAI - Embedded Systems and Artificial Intelligence Group Dept. of Physical Sciences, Mathematics and Computing Universidad CEU Cardenal Herrera, Valencia, Spain; Ghent University, BELGIUM

## Abstract

Nearly 1% of the global population has Epilepsy. Forecasting epileptic seizures with an acceptable confidence level, could improve the disease treatment and thus the lifestyle of the people who suffer it. To do that the electroencephalogram (EEG) signal is usually studied through spectral power band filtering, but this paper proposes an alternative novel method of preprocessing the EEG signal based on supervised filters. Such filters have been employed in a machine learning algorithm, such as the K-Nearest Neighbor (KNN), to improve the prediction of seizures. The proposed solution extends with this novel approach an algorithm that was submitted to win the third prize of an international Data Science challenge promoted by Kaggle contest platform and the American Epilepsy Society, the Epilepsy Foundation, National Institutes of Health (NIH) and Mayo Clinic. A formal description of these preprocessing methods is presented and a detailed analysis in terms of Receiver Operating Characteristics (ROC) curve and Area Under ROC curve is performed. The obtained results show statistical significant improvements when compared with the spectral power band filtering (PBF) typical baseline. A trend between performance and the dataset size is observed, suggesting that the supervised filters bring better information, compared to the conventional PBF filters, as the dataset grows in terms of monitored variables (sensors) and time length. The paper demonstrates a better accuracy in forecasting when new filters are employed and its main contribution is in the field of machine learning algorithms to develop more accurate predictive systems.

## Introduction

An estimation of the World Health Organization mention that about 50 million people around the world have Epilepsy [1]. Thus, epilepsy is a common neurological disorder affecting nearly 1% of the global population. An epileptic seizure starts with a storm of abnormal electrical activity in the brain. As it is stated in [2], this activity usually begins in one or two specific brain regions and can then expand to other parts of the brain. It can cause inconveniences in movement, sensation, mood and mental function. At worst cases, where a severe seizure is occurring, the person may have convulsions and lose consciousness. Such situation may become a terrible problem that disrupts the daily activity of the person who suffers from this disease.

Pharmacotherapy with anti-epileptic drugs is the keystone of epilepsy treatment, but 20–40% of patients continue having seizures despite medications [3–5]. People with less severe level of epilepsy also appeal to medicate themselves excessively due to the constant threat and fear of an unexpected seizure. Hence, the possibility of forecasting seizures with an acceptable confidence level, could substantially improve the treatment of the epilepsy and thus the lifestyle of the people suffering from this problem [3, 6].

Other methods followed by neurosurgeons [2] sometimes appeal to cut away the pieces of brain tissue where the seizures originate, but in the past decade they have had another solution through the implant of neurostimulators. Those devices send pulses of electricity, through the nervous system, to prevent such electrical storms in the brain. But again, too many electrical pulses in the brain could not be the best solution, as the overmedication, and it continues being necessary to study the brain’s electrical activity to find accurate patterns of seizures in order to forecast them. A precise seizure prediction system could allow to patients to abstain risky activities, relax their level of anxiety or avoid taking unnecessary medication [6].

Some studies have proved the feasibility of forecasting human and canine epileptic seizures in naturally occurring epilepsy using long recording of electroencephalogram (EEG). As it is stated in [7–9] naturally occurring canine epilepsy is an excellent model for human epilepsy.

EEG is a multichannel recording of the brain’s electrical activity. EEG electrodes are located on the scalp or invasively in the brain (intracranial EEG, iEEG). As a result of greater proximity to neural activity, iEEG has a higher spatial resolution and signal-to-noise ratio than scalp EEG, thus it is more valuable for epilepsy research [10].

One of the most useful features extracted of the iEEG signal is the spectral power in different frequency bands, as for example (*δ* (0.1-4 Hz), *θ* (4-8 Hz), *α* (8-12 Hz), *β* (12-30 Hz), low-*γ* (30-70 Hz) and high-*γ* (70-180 Hz)), which were used in [3] for prediction of canine epileptic seizures classifying preictal (prior to seizure) and interictal (between seizures) states of the individual. From now on, this preprocessing method with the average of the frequencies within these ranges is called Spectral Power Band Filter (PBF). Several studies sustain the hypothesis of an existing preictal state, which is associated with distinctive iEEG waveforms and spectral patterns [3]. This preprocessing technique is unsupervised, i.e., the procedure does not use the class labels of the training set. It must be said that there exist supervised preprocessing techniques used in other frameworks, for example Common Spatio-spectral Pattern (CSP) [11] or Kernel Fisher Discriminant (KFD). In general, these feature extraction techniques are used to locate relevant channels for a neurological state [12, 13].

National Institutes of Health (NINDS), the Epilepsy Foundation, the American Epilepsy Society and Mayo clinic sponsored an International data science competition known as “American Epilepsy Society Seizure Prediction Challenge” at the prestigious Kaggle platform. Its goal was to identify the best model for discriminating preictal vs interictal iEEG clips collected from dogs and persons. The ESAI research group participated, winning the third prize among more than 505 teams, coming from the most relevant universities and specialized enterprises from all over the world.

Our algorithm [14] considered as features, the spectral power in the six frequency bands and some other statistics. These were preprocessed by means of PCA (Principal Component Analysis) and ICA (Independent Component Analysis), and finally modeled with a combination of Artificial Neural Networks (ANN) and K-Nearest Neighbor (KNN) machine learning algorithms. In the present paper, the objective is to demonstrate how preprocessing the FFT (Fast Fourier Transform) signal, with a new approach, using supervised filters provides a much better outcome than conventional spectral power band filters or PBF. The machine learning technique selected to perform the comparison has been KNN, since the only parameter to tune is the number of neighbors. Nevertheless, it was compared with other machine learning algorithms to check whether similar conclusions can be drawn for such algorithms.

Consequently, the present study explores a new method of preprocessing the iEEG signal, based on the algorithm that was submitted to the competition. Such a new approach improves the final quality and performance of some learning machine techniques to detect preictal state, compared with other alternatives. The result of the study demonstrates that the bigger and more complex the volume of data acquired from the brain, the better performance for the methods we propose to detect these preictal states on the subjects under study, being the techniques presented in this paper useful for situations close to a big data problem. Next, it is described the dataset employed for the present study provided by Mayo Clinic and the basic formulae to preprocess the iEEG signal. Then, the machine learning algorithm and the ROC curve analysis are depicted describing the results of the comparison between several machine learning algorithms. Finally, discussion and conclusions state the reason why we consider the supervised filters proposed could improve epilepsy seizure forecasting and its reproducibility in other studies.

## Materials and methods

This section presents a description of the dataset available at Kaggle which have been used to validate different preprocessing algorithms. The description and formalization of these algorithms are thoroughly developed in the rest of this section. An implementation of the algorithm is available at the repository https://github.com/fjmalmaraz/supervised-filters for reproducibility.

### Dataset

The dataset, used for the challenge was provided by Mayo Clinic and it has been validated in several studies [3, 6, 9]. It was uploaded to the Kaggle platform and it is open to anyone who wants to employ it [15]. It is organized, as Intracranial EEG (iEEG) data clips, in folders containing training and testing data for each human or canine subject. There are data from five dogs and two persons. The training data is organized into ten-minute iEEG clips labeled “Preictal” for seizure data segments, or “Interictal” for non-seizure data segments. Training data segments are numbered sequentially, whereas testing data are in random order.

Preictal training and testing data segments were supplied covering one hour prior to seizure with a five-minute seizure temporal window (i.e. from 1:05 to 0:05 before seizure onset) This pre-seizure window ensures that: 1) seizures could be predicted with enough warning to allow administration of fast-acting medications, and 2) any seizure activity before the annotated onset that may have been missed by the epileptologist would not affect the outcome of the competition.

Similarly, one-hour sequences of interictal ten-minute data segments were also provided. The interictal data were chosen randomly from the full data record, with the restriction that interictal segments be as far from any seizure as can be practically achieved, to avoid contamination with preictal or postictal signals. In the long duration canine recordings it was possible to maintain a restriction of one week before or after a seizure. However, in the human recordings (which may be less than a week in total duration) interictal data was restricted to be more than four hours before or after any seizure.

iEEG data was recorded from five dogs with the naturally occurring epilepsy using an ambulatory monitoring system [3, 9]. The iEEG was sampled from 16 intracranial electrodes at 400 Hz, and recorded voltages were referenced to the group average. These are long duration recordings, spanning multiple months up to a year and recording up to a hundred seizures in some dogs. The dogs were housed at the veterinary hospitals at the University of Minnesota and University of Pennsylvania.

For the human patients, the iEEG was sampled from 15 electrodes at 5000 Hz, the recorded voltages were referenced to an electrode outside the skull; monitoring period for was up to a week. The epilepsy patients, who underwent the iEEG monitoring, were reviewed at Mayo Clinic Rochester. Interictal data segments were chosen at random, within the restrictions commented above, for both canine and human subjects. Table 1 depicts characteristics of recorded data and clip selection:the division of testing and training data clips and subjects’ characteristics about their monitoring sampling rate, hours of recorded data, seizures and lead seizures. Lead seizures are defined as seizures occurring without a preceding seizure for a minimum of 4h. The data provided was supervised by epileptologists of the prestigious institutions aforementioned. A complete description is provided in [9].

**Table 1:** Data characteristics for the Kaggle.com seizure forecasting contest. Source: [9].

Subject	Sampling rate (Hz)	Recorded data (h)	Seizures	Lead Seizures	Training clips (% interictal)	Testing clips (% interictal)
Dog 1	400	1920	22	8	504 (95.2)	502 (95.2)
Dog 2	400	8208	47	40	542 (92.3)	1000 (91.0)
Dog 3	400	5112	104	18	1512 (95.2)	907 (95.4)
Dog 4	400	7152	29	27	901 (89.2)	990 (94.2)
Dog 5	400	5616	19	8	480 (93.8)	191 (93.7)
Patient 1	5000	71.3	5	4	68 (73.5)	195 (93.9)
Patient 2	5000	158.5	41	6	60 (70.0)	150 (90.7)

The dataset, used for the competition, is still available at the Kaggle platform [15]. Thus, it is possible to continue exploring and improving the work done during the challenge. The Kaggle competition was based on the approach of long recording dataset available to explore different learning machine techniques in order to develop an accurate algorithm to forecast seizures.

### Overall risk of the optimal prediction for features

Generally, the success of any forecasting algorithm highly depends on an adequate selection of features, which is very often performed according to the technical expertise on the area. Nevertheless, signal or image processing generates such a huge volume of information that it is not the most efficient way just to rely on the experts’ opinion to tune the feature extraction, being convenient some automatic detection of high quality features.

The following approach is based on the concept of the overall risk of a predictor, which can be found for instance in [16]. Given some features, our objective is to determine the quality of the optimal predictor, assuming the theoretical distribution of the classes as known. Suppose there are two different classes, i.e. the variable *G* takes two possible values 0 and 1, and the value of this variable needs to be predicted by means of other variables which are represented by a random vector **X**. Let us suppose that the class-conditional probability density functions are known, being *f*_0_ and *f*_1_ the density functions for 0 and 1 classes respectively. If *p* is the prior probability of the class 1, a straightforward application of Bayes’ theorem provides the probability of belonging to the class 1:

$$\begin{array}{c} Pr\left(G=1|X=x\right)=\frac{f_{1}\left(x\right)p}{f(x)}=p_{x} \end{array}$$ (1)

where *f*(**x**) is the unconditional density function *f*(**x**) = *f*_1_(**x**)*p* + *f*_0_(**x**)(1 − *p*). The random variable *G* given that **X** = **x** is a Bernoulli random variable with the probability *p*_**x**_. A rational choice to fit the classifier is consider the regressor *α*(**x**) = *p*_**x**_ that minimizes the expected value of (*α*(**X**) − *G*)^2^, considering as the loss function *L*(**x**, *g*) = (*p*_**x**_ − *g*)^2^. Notice that other loss functions are possible, such as cross-entropy loss function, but they are out of the scope of this paper. The risk given a vector **x** of features is the expected value of the loss associated:

$$\begin{array}{c} R\left(x\right)=E\left[L\left(x,G\right)\right]=\sum_{g\in \{0,1\}}L\left(x,g\right)Pr\left(G=g|X=x\right)=p_{x}\left(1-p_{x}\right). \end{array}$$ (2)

Therefore, the risk is *R*(**x**) = *p*(1 − *p*)*f*_1_(**x**)*f*_0_(**x**)/*f*(**x**)^2^ and the overall risk is the integral of the risk multiplied by the unconditional density function is given by

$$\begin{array}{c} \left\langle R\right\rangle =\int_{R^{n}}p\left(1-p\right)\frac{f_{1}\left(x\right)f_{0}\left(x\right)}{f{\left(x\right)}^{2}}\;f\left(x\right)\;dx=p\;\left(1-p\right)\int_{R^{n}}\frac{f_{1}\left(x\right)\;f_{0}\left(x\right)}{f(x)}\;dx \end{array}$$ (3)

This value can be interpreted as the expected value of *p*(1 − *p*)*f*_1_(**Z**)/*f*(**Z**) where **Z** is a random variable whose density function is *f*_0_. The smaller the overall risk, the more useful the random vector of features **X** to predict the class *G*. If possible, the preprocessing of the features like the FFT signal for epileptic prediction should be performed in such a way that the overall risk of the new variables does not rise abruptly, being the potential prediction capability of our features lost.

### A lower bound of the overall risk

A set of features whose overall risk reaches the minimum of the optimal predictor would be the perfect choice for the problem of selecting the most useful variables to predict a binary classification. In most of the situations, a straightforward optimization of the overall risk would require long calculations if the number of original features is huge. Therefore, our proposal is less ambitious and, in this paper, we start with the selection of features trying to get a minimal value of a rough estimation of the lower bound of the overall risk. Our expectations are that this provides one of the best feature selection to perform a good prediction. This bound will be used as an insight to develop preprocessing algorithms for supervised classification. Let *f*_*max*_ be the maximum value of the unconditional density function, which can be considered a measure of the spread of the distribution. Therefore, *p*(1 − *p*)*E*[*f*_1_(**Z**)/*f*(**Z**)] is less than (*p*(1 − *p*)/*f*_*max*_)*E*[*f*_1_(**Z**)] where **Z** is a random variable with probability density function *f*_0_.

A kernel density estimator is used to approach *E*[*f*_1_(**Z**)] with

$$\begin{array}{c} E\left[\frac{1}{n\;h}\sum_{j=1}^{n}K\left(\frac{Z-Y_{j}}{h}\right)\right]=\frac{1}{n\;h}\sum_{j=1}^{n}E\left[K\left(\frac{Z-Y_{j}}{h}\right)\right]=\frac{1}{h}E\left[K\left(\frac{Z-Y}{h}\right)\right] \end{array}$$ (4)

where *K* is the kernel, i.e. positive function whose integral is one and **Y**_*j*_ are independent and identical variables whose density function is *f*_1_. For instance, *K*(**x**) = exp(−∥**x**∥^2^). Since the exponential function is convex, by Jensen’s inequality we have

$$\begin{array}{c} \exp(-E[∥\frac{Z-Y}{h}{∥}^{2}])\leq E[\exp(-∥\frac{Z-Y}{h}{∥}^{2})]=E\left[K\left(\frac{Z-Y}{h}\right)\right] \end{array}$$ (5)

In order to minimize the lower bound of the approximation to the variance of the prediction, we have to maximize $E[∥\frac{Z-Y}{h}{∥}^{2}])$, since the function exp(−*x*) is decreasing. Therefore, after fixing a value for *h*, the lower bound of the variance is

$$\begin{array}{c} \exp(-E[∥\frac{Z-Y}{h}{∥}^{2}])/\left(h\cdot f_{max}\right) \end{array}$$ (6)

Taking this expression as an approach to the overall risk, the overall risk of our features decreases with the expected value of the square of the difference and with the unconditional variability given by *f*_*max*_. Following these ideas, a method to choose the variables is that the expected value of the squared difference of them must be as large as possible. This approach is developed in the next section.

### Projection with an optimal lower bound (SqD)

In order to perform a binary classification using a large number of features, such as FFT using long input windows, becomes necessary a data preprocessing prior to use any machine learning algorithm, summarizing all these features in a small vector. The simplest summary is a linear combination or mixture of variables. Geometrically speaking, a set of variables is going to be summarized by a number employing a linear projection *π*_**u**_ where **u** is a unit vector on the direction along the features are projected, i.e. the projection is a scalar product *π*_**u**_(**x**) = **u** ⋅ **x**.

We consider **Z** a random vector of features under the condition of belonging to the class *G* = 0 and, analogously, **Y** a random vector of features given *G* = 1. Our objective is to minimize the numerator of the lower bound and this is equivalent to maximize the objective function *E*[(*π*_**u**_(**Z**) − *π*_**u**_(**Y**))^2^] where **u** is a unit vector. Applying Lagrange’s multipliers to the objective function and restriction ∥**u**∥^2^ = 1, the vector **u** where the optimal solution is attained is a critical point of the function of **u** and λ,

$$\begin{array}{c} u^{T}\;E\left[\left(Z-Y\right){\left(Z-Y\right)}^{T}\right]{)]u+\lambda (1-∥u∥}^{2}). \end{array}$$ (7)

Vanishing the derivatives of this function, we got the equations to find the critical point

$$\begin{array}{c} 2E[\left(Z-Y\right){\left(Z-Y\right)}^{T}])]u-2\lambda u=0, \end{array}$$ (8)

for unit vector **u**. Therefore, the critical points of Eq (7) are the eigenvectors of the matrix *E*[(**Z** − **Y**)(**Z** − **Y**)^*T*^]. To get the projection *π*_**u**_, we have selected the eigenvector with the largest eigenvalue by means of the power iteration algorithm. One of the main drawbacks to solve this equation is that the matrix can exceed the available computational resources. Taking into account that the data to calculate the matrix are distributed in multiple files, it is possible to apply this algorithm without storing the full matrix in memory. Let **Z**_*j*_ and **Y**_*j*_ be a sample from the training data. The initial vector **u**^(0)^ = **v**/∥**v**∥ where **v** is a vector such that all the components are 1. For the *k*th iteration, given the previous vector **u**^(*k*−1)^, we multiply this vector by an estimation of the matrix *E*[(**Z** − **Y**)(**Z** − **Y**)^*T*^])]

$$\begin{array}{c} v^{\left(k\right)}=\sum_{j}\left(\left(Z_{j}-Y_{j}\right)\cdot u^{\left(k-1\right)}\right)\left(Z_{j}-Y_{j}\right) \end{array}$$ (9)

and **u**^(*k*)^ is the normalized vector of **v**^(*k*)^, i.e. **u**^(*k*)^ = **v**^(*k*)^/∥**v**^(*k*)^∥.

### Alternative methods

Using a wider interpretation of the estimation of the lower bound, we are proposing several preprocessing methods based on the idea that the larger is the expected differences between two signals of distinct classes and the smaller is the unconditional variance, the better forecasting results are expected to get. Our alternatives consider the difference of the means (DM), the difference of the variance between the two clases (VAR), a linear combination which tries to trade off the variance and the mean (TVM) and a very simplified preprocessing method which consists in the difference of the square (DS). The last method winds up to be the more effective filters according to the experimental results.

#### Difference of the Mean(DM)

The most intuitive idea is to estimate a projection *π*_*u*_ such that the difference of projected vectors mean have the larger possible value. Hence, the objective function is *E*[*π*_**u**_(**Z**) − *π*_**u**_(**Y**)] = *E*[**u** ⋅ (**Z** − **Y**)]. This function is the scalar product with the vector difference of the mean *E*[(**Z** − **Y**)] ⋅ **u** and the maximum is attained at the point **u** = *E*[(**Z** − **Y**)]/∥*E*[(**Z** − **Y**)]∥. This preprocessing is straightforward and no iterative process is needed.

#### Variance (VAR)

Most of literature preprocessing methods consist in maximizing the variance to learn new discriminative features. For binary classification, some methods, like Common Spatial Patterns (CSP), maximize the generalized Rayleigh quotient whose solutions are generalized eigenvalues [11, 17, 18]. The main drawback of this method is that the covariance matrix must be computed and full stored in the computer. For a problem with a large number of features this procedute cannot be performed. The difference between the variances can be expected to have one of the classes with a large variance and the other class with a smaller variance. In this situation, the objective function is

$$\begin{array}{c} E\left[{\left(\pi_{u}\left(Z\right)-E\left[\pi_{u}\left(Z\right)\right]\right)}^{2}\right]-E\left[{\left(\pi_{u}\left(Y\right)-E\left[\pi_{u}\left(Y\right)\right]\right)}^{2}\right] \end{array}$$ (10)

being equals to

$$\begin{array}{c} E\left[\pi_{u}{\left(Z\right)}^{2}\right]-E\left[\pi_{u}{\left(Y\right)}^{2}\right]-E{\left[\pi_{u}\left(Z\right)\right]}^{2}+E{\left[\pi_{u}\left(Y\right)\right]}^{2} \end{array}$$ (11)

This is a quadratic function whose associated matrix is:

$$\begin{array}{c} S=E\left[ZZ^{T}\right]-E\left[YY^{T}\right]-E\left[Z\right]E{\left[Z\right]}^{T}+E\left[Y\right]E{\left[Y\right]}^{T}. \end{array}$$ (12)

This matrix *S* is symmetric. Therefore, the maximum of the quadratic form **u**^*T*^ *S***u** restricted to unit vectors **u** is the eigenvector associated to the largest eigenvalue. The largest eigenvalue can be calculated with the power iterative method based on successive multiplications by the matrix and division of the outcome by its norm. For a large scale data set, it is possible to perform multiplications of the matrix *S* by a vector without storing the matrix in main memory in a similar way as done in Eq (9).

#### Trade-off Variance-Mean (TVM)

Another proposal for objective function appears searching a trade off between the mean and the variance. Therefore, the objective function is the sum of square of the objective function for the mean and the variance difference. In this situation, the algorithm is to solve a quadratic program with the objective function

$$\begin{array}{c} E[{\left(\pi_{u}\left(Z\right)-\pi_{u}\left(Y\right)\right]}^{2}+Var\left(\pi_{u}\left(Z\right)\right)-Var\left(\pi_{u}\left(Y\right)\right), \end{array}$$ (13)

whose critical points are the eigenvectors of the following matrix

$$\begin{array}{c} E\left[ZZ^{T}\right]-E\left[YY^{T}\right]+\left(E\left[Y\right]-E\left[Z\right]\right)E{\left[Y\right]}^{T}+E\left[Y\right]{\left(E\left[Y\right]-E\left[Z\right]\right)}^{T}. \end{array}$$ (14)

The calculations are performed analogously to the previous sections.

#### Squared simplification of the trade-off (DS)

In the previous objective function, the expression can be simplified removing the terms which are depending on the expected values of the variables. Hence, the objective function becomes *E*[(*π*_**u**_(**Z**))^2^ − (*π*_**u**_(**Y**))^2^] whose interpretation is that the projection is maximizing the difference of the squares of the variables between the classes. The objective function can be rewritten as a quadratic form where the matrix of the quadratic form is the tensor product of both variables multiplied by themselves.

$$\begin{array}{c} E\left[{\left(uZ\right)}^{2}-{\left(uY\right)}^{2}\right]=u^{T}\left(E\left[ZZ^{T}\right]-E\left[YY^{T}\right]\right)u \end{array}$$ (15)

During the following sections we are going to compare all these methods with several machine learning algorithms, being the focus on KNN.

### K-Nearest neighbors classifier

K-Nearest Neighbors (KNN) models were chosen because of their non-parametric nature, since the training data are the model itself. A KNN model has one sole hyper-parameter that is the number of neighbors, denoted by *K*. The *K* neighbors given by KNN were transformed into probabilities following the implementation given in APRIL-ANN toolkit [19], similar to [20]. It basically computes a posterior probability by normalizing the exponential of the negative distances, following this equation:

$$\begin{array}{c} Pr\left(G=1|x\right)=\frac{\sum_{y\in K_{1}}\exp\left({-∥y-x∥}_{2}^{2}\right)}{\sum_{y\in K}\exp\left({-∥y-x∥}_{2}^{2}\right)} \end{array}$$ (16)

where **x** is an input sample, K is the set of *K*-neighbors of **x**, $K_{1}$ is the intersection of K and the class *G* = 1. The adequate value of *K* and the computation of probabilities using distances reduce the impact of over-fitting in the KNN model.

The hyper-parameter *K* was estimated during Kaggle challenge according to the performance in a cross-validation scheme and the public AUC, using for this estimation PBF preprocessing for each channel. The number of blocks has been set to the number of seizures recorded for every particular subject, leaving one seizure out for validation. A value *K* = 40 neighbors achieved good AUC for cross-validation and the best AUC at Kaggle public test partition. Since the hyper-parameter *K* = 40 has been chosen with PBF preprocessing, proposed preprocessing techniques could have a different optimal values for *K*. Therefore, our conclusions would be expected to be biased in favour of PBF.

### Prediction procedure

The goal of the system is to produce a posterior probability for preictal class given a file with 10 minutes of iEEG recording. The system is a pipeline of several methods divided in two stages: preprocessing and classification. Such system is an adaptation of the one presented in [6] and it is available for reproducibility issues at [14].

**Fig 1:**
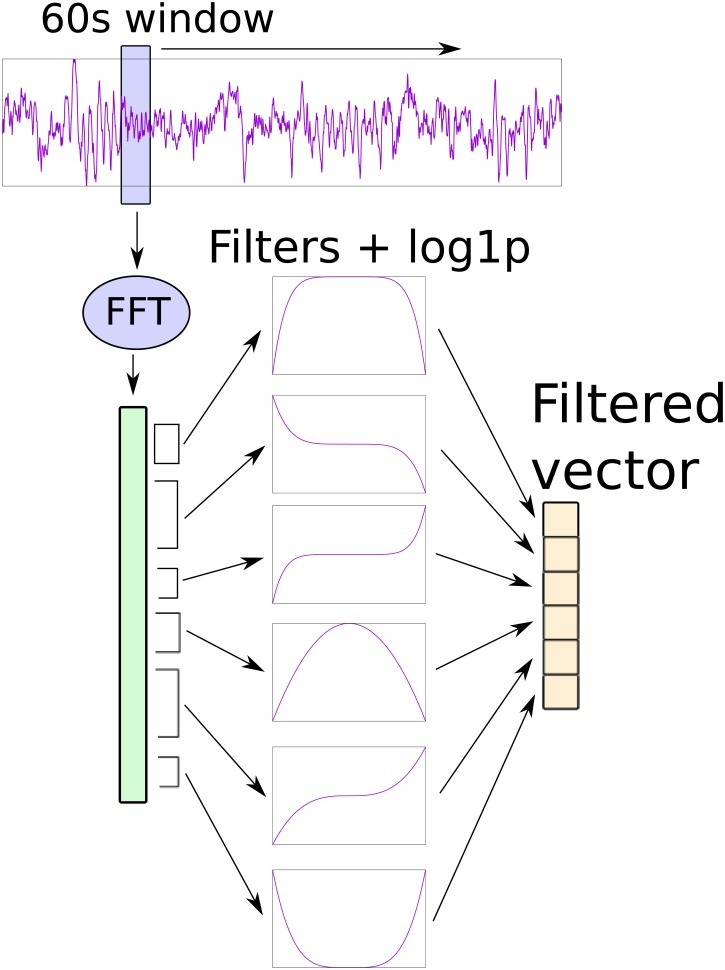
Preprocessing stage diagram. A sliding window is used to extract 60-second windows which are processed by FFT to generate a large vector. This vector is preprocessed with 6 filters located at the corresponding 6 bands (*δ*, *θ*, *α*, *β*, low-*γ*, high-*γ*). These filters have been previously calculated with the preprocessing methods described in this paper. Finally the output of the filters is log-compressed with the function log1p (Natural logarithm of 1 + *x*, element-wise). This procedure is repeated for each available iEEG channel.

Starting at preprocessing stage, 1-minute Hamming windows are generated with 30 seconds of overlap from 10-minute iEEG signal, FFT transformed, log compressed and filtered according to the methods described in this paper. PBF filter is used in literature to summarize the FFT in six features per channel, according to the frequency bands *δ* (0.1-4 Hz), *θ* (4-8 Hz), *α* (8-12 Hz), *β* (12-30 Hz), low-*γ* (30-70 Hz) and high-*γ* (70-180 Hz). For each band, PBF computes an uniform average of all FFT bins corresponding to the frequencies within the band. All other supervised filtering methods proposed in this paper summarize the FFT into the same six bands. Let us remark that, instead of an arithmetic mean, these methods compute a weighted average of the FFT bins related with the frequencies of each band. These weights are estimated following the procedures described in the previous section, highlighting the most important frequencies in order to improve discrimination between classes. Fig 1 shows how preprocessing stage is performed.

The second stage consists in the classification of an input file given its extracted features. The classifier produces a posterior probability of preictal state for each time slice of FFT sliding window. Due to there are 19 time intervals for every 10-minute input file, the model produces 19 posterior probabilities for each input file. Thus, in order to compute preictal posterior probability for one file it is required to aggregate these 19 probabilities. In order to increase sensitivity, these posterior probabilities are aggregated into one value following a geometric mean but complementing each input probability. Finally, complement operation is repeated to obtain the posterior preictal probability for the given input file. Eq (17) formalizes this process:

$$\begin{array}{c} Pr\left(\text{preictal}|x\right)=1-\sqrt[{n}]{\prod_{t=1}^{n}\left(1-Pr(\text{preictal}|x_{t})\right)} \end{array}$$ (17)

where *n* = 19 is the number of window slices, **x** is a matrix with *n* feature vectors and *p*(preictal|**x**_*t*_) is posterior probability of KNN for feature vector *t* computed using Eq (16).

## Results-ROC analysis

Using the Receiver Operating Characteristic (ROC) curve a quantitative assessment of the model can be obtained and therefore it is possible to represent the trade-off between sensitivity and specificity of the underlying model. Thus, an optimal point can be found in the curve in order to decide when a sample should be classified as true or false by the model. The ROC curve is created by evaluating the class probabilities for the model across a continuum of thresholds.

**Fig 2:**
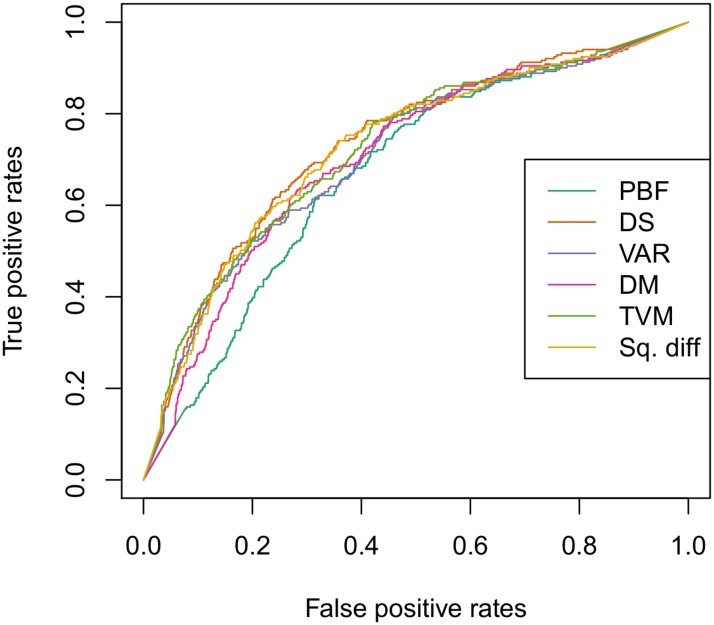
ROC curves for the preprocessing methods. Following the notation in the paper: PBF (dark green), DS (orange), VAR (blue), DM (pink), TVM (light blue), Sq.diff (yellow) for all the test set (public+private) given by the Kaggle contest.

In Fig 2, the ROC curves of the KNN predictions with the preprocessing methods are compared. The greater area under the curve, the better the model is for the prediction. Accordingly, Kaggle competition states to use the Area Under Curve (AUC) as a measure of model goodness. The values calculated by the Kaggle platform for the preprocessing methods in the public and private set are shown in Table 2, using KNN as machine learning algorithm. All the proposed methods improve considerably the performance of the overall system with respect to the PBF, in both public and private sets as shown in Table 2.

**Table 2:** Public and private scores in the American Epilepsy Society Seizure Prediction Challenge for KNN with 40 neighbors for the preprocessing methods.

Method	PBF	DS	VAR	DM	TVM	SqD
Public	0.67589	0.73958	0.71413	0.71106	0.73047	0.73967
Private	0.66939	0.72846	0.70507	0.70198	0.71593	0.71347

With the purpose of answering whether or not the differences seen in Fig 2 are due to chance, Delong’s method [21] has been used to test AUC differences between standard PBF preprocessing and our methods. The p-values for these testings are shown in Table 3 and it can be observed that all the proposed methods got a significant difference compared with the prediction using the conventional standard band filters (PBF). However, we have not detected any significant difference between the supervised preprocessing methods proposed in this paper, except for the DM method which is the poorest method of the proposed ones. Moreover, the AUC differences have also been tested for every individual, those whose volume of data is large enough, have undergone a much better performance with the supervised preprocessing methods as it is shown in Table 3.

**Table 3:** Comparison with the standard band filters (PBF) and the preprocessing techniques.

	DS	VAR	DM	TVM	SqD	Features	Samples
All	2.2e-08‡	0.00034‡	0.0084‡	2.6e-06‡	3.2e-06‡	-	4 057
Dog 1	0.1	0.14	0.35	0.033♢	0.45	262 144	504
Dog 2	0.61	0.00029♢	8.2e-06♢	0.024♢	0.7	262 144	542
Dog 3	3e-12‡	4.5e-09‡	1e-07‡	3.2e-09‡	9e-08‡	262 144	1512
Dog 4	0.0025‡	0.033‡	0.0085‡	0.024‡	0.21	262 144	901
Dog 5	0.13	0.16	0.12	0.64	0.12	245 760	480
Patient 1	0.22	0.98	0.68	0.48	0.18	3 932 160	68
Patient 2	0.025‡	0.028‡	0.6	0.00016‡	0.08	6 291 456	50

For every cell the p-value is pointed out, being the null hypothesis that the preprocessing method in the column has the same AUC as PBF preprocessing method. The number of features for an individual is the product of the number of frequencies and the number of channels. The column Samples is the number of observations for every individual in the training set. If the proposed preprocessing method is significantly better that PBF, the cell is marked with ‡. The case of the difference being favourable to PBF is marked with ♢.

A more detailed analysis can be performed comparing AUC between subjects for each of the proposed methods and the PBF method. Two random partitions of all Kaggle test data have been created to this purpose, using same Kaggle proportions which are 40% of the data for validation and 60% for test. Due to the fact that hyperparameters have been chosen with the previous partition, AUC calculations could be slightly optimistic. Nevertheless, hyperparameters have not been chosen according to performance with the proposed filters, hence AUC calculations with these filters are expected to be less optimistic than with PBF. Tables 4 and 5 show the results of AUC for the different individuals and for the total data (pool column), which is evidence of generalization of these techniques. The pool column demonstrates that our filters improve the AUC results in the validation and test sets. All the proposed filters got an AUC result above 0.7 whereas conventional filters (PBF) are around 0.67 in the best case. Such results are coherent with the p-value of all the dataset in Table 3, which proves that there is a significant improvement with these methods.

**Table 4:** AUC results for the validation set.

Model	Filter	Dog 1	Dog 2	Dog 3	Dog 4	Dog 5	Pat. 1	Pat. 2	Pool
KNN	PBF	**0.804**	0.807	0.547	0.737	0.517	0.611	0.458	0.663
	DS	0.750	0.809	0.716	**0.796**	0.466	0.737	0.616	**0.732**
	VAR	0.738	0.724	0.672	0.779	0.479	0.691	0.537	**0.699**
	DM	**0.804**	0.710	**0.765**	0.774	**0.524**	0.672	0.378	**0.706**
	TVM	0.726	0.756	0.675	0.769	0.493	0.691	**0.675**	**0.708**
	SqD	0.777	**0.812**	0.680	0.767	0.473	**0.754**	0.593	**0.720**

For this comparison, AUCs have been calculated for the validation set which is a sample of the 40% of test data available at Kaggle. The bold-faced number is the best performance for the individual got with the different classifier systems. Average of subjects AUC is shown at column Average. Pool column shows AUC computed after union of all subject predictions, as done by Kaggle framework.

**Table 5:** AUC results for test set.

Model	Filter	Dog 1	Dog 2	Dog 3	Dog 4	Dog 5	Pat. 1	Pat. 2	Pool
KNN	PBF	**0.885**	**0.756**	0.673	0.777	**0.585**	0.711	0.470	0.679
	DS	0.865	0.741	0.796	0.823	0.462	0.720	0.577	**0.735**
	VAR	0.814	0.686	0.796	0.804	0.462	0.647	0.643	**0.716**
	DM	0.763	0.651	0.786	**0.835**	0.382	0.634	0.463	**0.705**
	TVM	0.798	0.713	**0.805**	0.811	0.530	0.703	**0.695**	**0.733**
	SqD	0.866	0.742	0.768	0.801	0.453	**0.722**	0.571	**0.728**

For this comparison, AUCs have been calculated for the test set which is a sample of the 60% of test data available at Kaggle.

Moreover, results from the different subjects demonstrate that for dogs 3 and 4 and the humans the proposed filters always improve the AUC results in both sets. This is due to Dogs 3 and 4 have many more samples than Dogs 1 and 2. On the other side, although humans have less samples they have been sampled at a rate of 5000 Hz, i.e. much more information for both individuals. Fewer samples has Dog 5 and thus obtains very bad results in forecasting introducing some noise when evaluating the overall dataset. To mention that using a validation test allows us to select preprocessing algorithms with good performance in the test set.

In conclusion, the standard filters seem to be comparable to the supervised filters when the volume of data is limited or reduced. Nevertheless, our conjecture is that there exists an important improvement when data available for training grows substantially.

As seen in Table 3, the proposed methods show statistical significant (for *α* = 0.05) improvements when compared with PBF filter taken as the baseline. The positive behavior of this preprocessing motivates a deeper comparison of the methods using any other classifier algorithms, in order to check if they were able to get similar improvements to KNN. The most used algorithms among Kaggle challenges have been chosen to predict and perform a comparison with the preprocessing technique: Support Vector Classifier (SVC), Gradient Boosting Classifier (GBC), Random Forest (RF) and Logistic Regression (LR). The hyper-parameters for these algorithms have been chosen to be similar according to the best performance values used by other participants during the Kaggle competition [9], since AUC calculations are done using the new partition, these AUC calculations could be slightly optimistic. The AUC values of these algorithms are shown in Table 6. The DS filter has been selected as it obtained in general a better performance than the others. It is observed that under no circumstances is KNN competitive with other machine learning algorithms using conventional filters as shown in Table 6. Not only is KNN performance improved using DS filter, but also it becomes competitive with the rest of the algorithms.

**Table 6:** Comparison with PBF and DS preprocessing methods using different learning machine algorithms.

Model	Filter	Dog 1	Dog 2	Dog 3	Dog 4	Dog 5	Pat. 1	Pat. 2	Pool
KNN	PBF	0.885	0.756	0.673	0.777	0.586	0.711	0.470	0.679
	DS	0.865	0.741	0.796	0.823	0.462	0.720	0.577	0.735
SVC	PBF	0.924	0.870	0.759	0.845	0.263	0.682	0.646	0.734
	DS	0.888	0.827	0.783	0.764	0.292	0.824	0.617	0.732
GBC	PBF	0.882	0.665	0.728	0.853	0.258	0.822	0.774	0.709
	DS	0.877	0.642	0.681	0.868	0.193	0.832	0.373	0.691
RF	PBF	0.848	0.672	0.623	0.854	0.514	0.778	0.812	0.695
	DS	0.858	0.652	0.671	0.863	0.419	0.814	0.341	0.694
LR	PBF	0.816	0.777	0.690	0.865	0.439	0.681	0.436	0.648
	DS	0.683	0.717	0.664	0.876	0.450	0.598	0.425	0.626

For this comparison, AUCs have been calculated for the test set which is a sample of the 60% of test data available at Kaggle.

This behavior suggests a tight coupling between KNN space tessellation and the proposed preprocessing methods. It can be explained informally as a result of the proposed lower bound for variance, that follows Eq (6) employing a kernel density estimator whose probability estimation resembles Eq (16).

Table 7 shows confidence intervals of AUC for the different machine learning methods and three subsets of frequency bands. The first subset consists in the prediction using all the six bands, the second subset includes the band with the lowest frequency and the third one corresponds to the prediction of the band with only the highest frequencies. Even though our preprocessing method is able to summarize a large number of variables, the predictions with the highest band have not a better performance than the standard method (PBF).

**Table 7:** Confidence interval for pool AUC with all the individuals.

		All frequency bands (0.1-180 Hz)		*δ* (0.1-4 Hz)		high-*γ* (70-180 Hz)	
Model	Filter	AUC Validation	AUC Test	AUC Validation	AUC Test	AUC Validation	AUC Test
KNN	PBF	0.663 ± 0.051	0.679 ± 0.044	0.673 ± 0.052	0.700 ± 0.044	0.608 ± 0.048	0.571 ± 0.048
	DS	0.732 ± 0.049	0.735 ± 0.043	0.677 ± 0.049	0.698 ± 0.043	0.600 ± 0.053	0.552 ± 0.048
SVC	PBF	0.744 ± 0.059	0.734 ± 0.050	0.576 ± 0.057	0.625 ± 0.044	0.719 ± 0.054	0.692 ± 0.049
	DS	0.740 ± 0.052	0.732 ± 0.047	0.610 ± 0.055	0.645 ± 0.043	0.566 ± 0.051	0.619 ± 0.042
GBC	PBF	0.709 ± 0.050	0.709 ± 0.046	0.679 ± 0.055	0.718 ± 0.047	0.693 ± 0.050	0.645 ± 0.045
	DS	0.688 ± 0.055	0.691 ± 0.045	0.670 ± 0.053	0.710 ± 0.045	0.523 ± 0.061	0.577 ± 0.047
RF	PBF	0.670 ± 0.060	0.695 ± 0.045	0.662 ± 0.054	0.696 ± 0.044	0.653 ± 0.053	0.629 ± 0.042
	DS	0.674 ± 0.057	0.694 ± 0.047	0.640 ± 0.055	0.687 ± 0.043	0.556 ± 0.057	0.588 ± 0.045
LR	PBF	0.667 ± 0.050	0.648 ± 0.046	0.639 ± 0.057	0.658 ± 0.046	0.577 ± 0.060	0.566 ± 0.046
	DS	0.633 ± 0.049	0.626 ± 0.045	0.625 ± 0.054	0.637 ± 0.043	0.526 ± 0.061	0.564 ± 0.046

## Discussion

**Fig 3:**
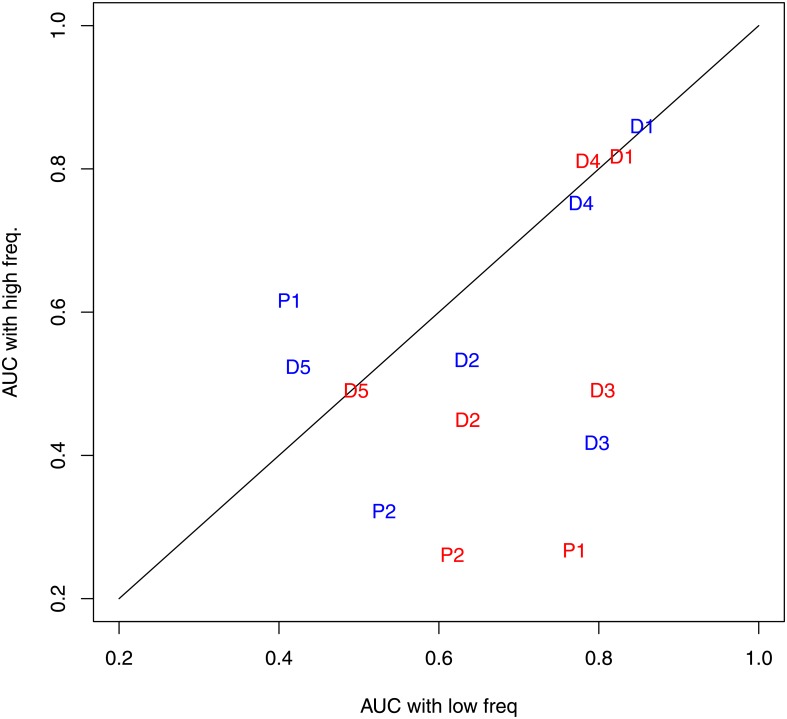
Comparison between AUC for individuals, preprocessing methods and the highest and the lowest frequency group. Every panel is a comparison between all the bands, only *δ* (0.1-4Hz) or only high-*γ* (70-180Hz). The red points correspond to the AUC got by the DS preprocessing and the black points by the PBF method. Every point is labeled according to the individual D1 (Dog 1), D2 (Dog 2), D3 (Dog 3), D4 (Dog 4), D5 (Dog 5), P1 (Patient 1) and P2 (Patient 2). Most of the points are in the lower bisector of the first quadrant, pointing out that the lowest frequency band is more useful than the highest frequency band with respect to forecasting seizures.

In [22] a Kernel Fisher Discriminant (KFD) analysis was performed determining that high gamma power with the raw signal is not found between the most discriminant features for most of the cases. Even though, their conclusion differs for time-differential signal, according to these authors raw signal is dominated by small changes in low frequencies. With the purpose of verifying whether low frequencies of the signal are more useful for predictions and the effect of preprocessing techniques, AUC values for each subject have been calculated with predictions based on KNN. Moreover, it was considered only the lowest and the highest frequencies against PBF and DS preprocessing techniques, being compared in Fig 3. Whereas there is an obvious improvement with low frequencies using DS preprocessing, there is not such a gain if only the highest frequency band is considered. Please notice that two of the individuals (Dog 3 and Dog 4) with the largest volume of information got a better performance with DS for the highest frequency band. This suggests that a dataset with subjects with a larger volume of information could give some pieces of evidence that this method could also be effective for high-*γ* frequencies.

In this paper, we have provided evidence that several preprocessing methods improves KNN. These algorithms do not have the same improvement using other machine learning algorithms, finding tailored supervised preprocessing could be a promising scope for future work to improve prediction techniques.

## Conclusions

The present paper extends the work carried out in detecting preictal states using an algorithm that was submitted to win the third prize of an international research challenge proposed by the American Epilepsy Society, the Epilepsy Foundation, National Institutes of Health (NIH) and Mayo Clinic through the Kaggle platform. It has been evaluated within this study if it is possible to design and develop the fundamental equations to obtain a new method of preprocessing the iEEG signal.

The idea is to find if an alternative iEEG supervised signal preprocessing, different from the conventional filters bank (PBF), could improve the forecasting results of a machine learning algorithm as the KNN. Accordingly, to remark that our main contribution is in the field of machine learning and statistics and not the clinical one, as our objective is to develop new improved predictive systems with better accuracy. Results seem highly promising as the performance, robustness and quality of the predictions have been improved, mainly when the data is getting bigger and bigger. Such behavior opens the usefulness of the method if we think for the future as a big data problem. That is, if the iEEG data stream coming from the brain is continuous and has to be processed in real-time. Although this concept is not the focus of the present study, it is true that when data clips had more variables (i.e. brain sensors) and more recording duration at a higher sampling rate, the quality of the prediction improved considerably, being able to have learning machine algorithm to detect preictal states in long recording iEEG is a first step for seizure forecasting.

A concern and indeed a challenge is the necessary evolution from some conventional techniques to the new framework given by the Big Data due to the increasing capabilities, for example, of real-time monitoring of body health indicators. In our opinion the treatment of such massive datasets with softer techniques than the conventional ones will be required by the new juncture. This opens a new task for us in evaluating the performance and suitability of our work in other areas.

## References

[1] World Health Organization. Epilepsy; 2015. WHO Fact Sheet 999.

[2] Denison T, Morris M, Sun F. Building a bionic nervous system. Spectrum, IEEE. 2015 2;52(2):32–39. 10.1109/MSPEC.2015.7024509

[3] Howbert JJ, Patterson EE, Stead SM, Brinkmann B, Vasoli V, Crepeau D, et al Forecasting seizures in dogs with naturally occurring epilepsy. PloS one. 2014;9(1):e81920 10.1371/journal.pone.0081920 24416133 PMC3885383

[4] Kwan P, Brodie MJ. Early identification of refractory epilepsy. New England Journal of Medicine. 2000;342(5):314–319. 10.1056/NEJM200002033420503 10660394

[5] Murray CJ, Lopez AD, et al Global comparative assessments in the health sector: disease burden, expenditures and intervention packages. Geneva: World Health Organization;

[6] Brinkmann BH, Patterson EE, Vite C, Vasoli VM, Crepeau D, Stead M, et al Forecasting seizures using intracranial EEG measures and SVM in naturally occurring canine epilepsy. PloS one. 2015;10(8):e0133900 10.1371/journal.pone.0133900 26241907 PMC4524640

[7] Potschka H, Fischer A, Rüden EL, Hülsmeyer V, Baumgärtner W. Canine epilepsy as a translational model? Epilepsia. 2013;54(4):571–579. 10.1111/epi.12138 23506100

[8] Patterson EE. Canine epilepsy: an underutilized model. ILAR Journal. 2014;55(1):182–186. 10.1093/ilar/ilu021 24936038

[9] Brinkmann BH, Wagenaar J, Abbot D, Adkins P, Bosshard SC, Chen M, et al Crowdsourcing reproducible seizure forecasting in human and canine epilepsy. Brain. 2016;139(6):1713–1722. 10.1093/brain/aww045 27034258 PMC5022671

[10] Korshunova I. Epileptic seizure prediction using deep learning. Universiteit Gent Belgium; 2015.

[11] Qi F, Li Y, Wu W. RSTFC: A Novel Algorithm for Spatio-Temporal Filtering and Classification of Single-Trial EEG. Neural Networks and Learning Systems, IEEE Transactions on. 2015 12;26(12):3070–3082. 10.1109/TNNLS.2015.2402694 25730834

[12] Kodipaka S, Vemuri BC, Rangarajan A, Leonard CM, Schmallfuss I, Eisenschenk S. Kernel fisher discriminant for shape-based classification in epilepsy. Medical Image Analysis. 2007;11(1):79–90. 10.1016/j.media.2006.10.002 17157051 PMC2267687

[13] Lemm S, Blankertz B, Curio G, Müller KR. Spatio-spectral filters for improving the classification of single trial EEG. Biomedical Engineering, IEEE Transactions on. 2005;52(9):1541–1548. 10.1109/TBME.2005.851521 16189967

[14] Zamora-Martínez F, Muñoz-Almaraz FJ, Botella-Rocamora P, Pardo J. Seizure prediction system of ESAI-CEU-UCH team. Universidad CEU Cardenal Herrera; 2015. https://github.com/ESAI-CEU-UCH/kaggle-epilepsy.

[15] Kaggle Inc. American Epilepsy Society Seizure Prediction Challenge; 2015. https://www.kaggle.com/c/seizure-prediction.

[16] Duda RO, Hart PE, Stork DG. Pattern classification. John Wiley & Sons; 2012.

[17] Koles ZJ, Lazar MS, Zhou SZ. Spatial patterns underlying population differences in the background EEG. Brain Topography. 1990;2(4):275–284. 10.1007/BF01129656 2223384

[18] Parlett B. The Symmetric Eigenvalue Problem Society for Industrial and Applied Mathematics; 1998.

[19] Zamora-Martínez, F, España-Boquera, S, Gorbe-Moya, J, Pastor-Pellicer, J, Palacios-Corella, A. APRIL-ANN toolkit, A Pattern Recognizer In Lua with Artificial Neural Networks; 2014. https://github.com/pakozm/april-ann.

[20] Goldberger J, Roweis S, Hinton G, Salakhutdinov R. Neighbourhood Components Analysis In: Advances in Neural Information Processing Systems (NIPS). MIT Press; 2005 p. 513–520.

[21] DeLong ER, DeLong DM, Clarke-Pearson DL. Comparing the Areas under Two or More Correlated Receiver Operating Characteristic Curves: A Nonparametric Approach. Biometrics. 1988 9;44(3):837–845. 10.2307/2531595 3203132

[22] Park Y, Luo L, Parhi KK, Netoff T. Seizure prediction with spectral power of EEG using cost-sensitive support vector machines. Epilepsia. 2011;52(10):1761–1770. 10.1111/j.1528-1167.2011.03138.x 21692794

